# Report of SARS-CoV-2 JN.1 variant in Morocco

**DOI:** 10.1128/mra.00559-24

**Published:** 2024-08-20

**Authors:** Oumaima Bouddahab, Adil El Hamouchi, Safaa Aqillouch, Hicham Charoute, Rachid Noureddine, Achraf Aainouss, Hanâ Baba, Ahd Ouladlahsen, Jalal Nourlil, M'hammed Sarih, Abderrahmane Maaroufi, Mustapha Lkhider, Abdelhamid Barakat, Sayeh Ezzikouri

**Affiliations:** 1 Virology Unit, Viral Hepatitis Laboratory, Institut Pasteur du Maroc, Casablanca, Morocco; 2 Laboratory of Virology, Microbiology, Quality and Biotechnology/Ecotoxicology and Biodiversity, Faculty of Sciences and Techniques of Mohammedia, Hassan II University of Casablanca, Mohammedia, Morocco; 3 Centre de Séquençage Génomique, Institut Pasteur du Maroc, Casablanca, Morocco; 4 Research Unit of Epidemiology, Biostatistics and Bioinformatics, Institut Pasteur du Maroc, Casablanca, Morocco; 5 Laboratoire Morizgo d’Analyses Médicales, Casablanca, Morocco; 6 Service des Maladies Infectieuses, CHU Ibn Rochd, Casablanca, Morocco; 7 Medical Virology and BSL3 Laboratory, Institut Pasteur du Maroc, Casablanca, Morocco; 8 Service de Parasitologie et des Maladies Vectorielles, Institut Pasteur du Maroc, Casablanca, Morocco; 9 Laboratory of Genomics and Human Genetics, Institut Pasteur du Maroc, Casablanca, Morocco; DOE Joint Genome Institute, Berkeley, California, USA

**Keywords:** SARS-CoV-2, JN.1 variant, nasopharyngeal swab, Morocco, next-generation sequencing

## Abstract

In this study, we report the identification of the severe acute respiratory syndrome coronavirus 2 (SARS-CoV-2) JN.1 variant and the quasi-complete genomic sequencing of four clinical samples in Morocco. Nasopharyngeal swabs were obtained from four patients (one female, three males). The Illumina COVIDSeq Test was used for comprehensive genomic analysis.

## ANNOUNCEMENT

August 2023 witnessed the worldwide emergence of the novel severe acute respiratory syndrome coronavirus 2 (SARS-CoV-2) (*Coronaviridae*, *Betacoronavirus*) sublineage BA.2.86, which has over 30 mutations in its spike (S) protein (1, 2). By late 2023, BA.2.86 had evolved into JN.1 (BA.2.86.1.1), which had one more mutation (L455S) within the receptor-binding domain of spike protein and three other non-spike protein mutations (3). These changes strengthened immune evasion, thus enabling rapid global dissemination of JN.1 infection (4). Regular and systematic monitoring, as well as characterizing the genetic variability of emerging SARS-CoV-2 variants, is now necessary (5).

In this study, Illumina technology was used for quasi-complete genome sequencing of SARS-CoV-2. Between December 18, 2023 and January 5, 2024, nasopharyngeal swabs were collected from four patients at Ibn Rochd University Hospital Center and private clinics in Casablanca, Morocco. The median age was 73.5 years, with three men and one woman. One man with a heart disease and a hypertension died from SARS-CoV-2 complications. All samples tested positive for coronavirus disease 2019 (COVID-19) using the COVID-19 Speedy RT-PCR kit (PCL Inc., Republic of Korea) at Morizgo Laboratory and were then sent for sequencing to the Viral Hepatitis Laboratory at the Institut Pasteur of Morocco.

Viral RNA was extracted using the QIAamp Viral RNA Mini Kit (Qiagen, Germany). Libraries were prepared according to Illumina COVIDSeq protocol (Illumina Inc., USA). The cDNA was synthesized using RT-PCR and was amplified with two PCR reactions utilizing primer pools from COVIDSeq PCR Master Mixes 1 and 2. Following Integrated DNA Technologies (IDT) for Illumina-PCR Indexes Set 1-4 tagging and adaptor ligation, the PCR products were cleaned according to the manufacturers’ protocols. Subsequently, the tagging of these amplicons was carried out using PCR. By increasing the quantity of DNA available for sequencing, this step ensures that there is sufficient material to create a robust sequencing library. Pooled samples were quantified and normalized to 4 nM in 25 µL using a Qubit 2.0 fluorometer (Invitrogen Inc.). Paired-end sequencing with a high-throughput reagent kit and 2 × 150 read lengths on the NextSeq 2000 ensured optimal coverage.

The genetic test for COVIDSeq on BaseSpace Sequence Hub was analyzed by DRAGEN. BCL CONVERT changed FASTQ sequence to BCL , and then the initial output was assembled, filtered, and quality checked. DRAGEN COVID Lineage v4 alignment was performed with human control amplicon sequences together with SARS-CoV-2 reference genome NC_045512.1. Variant calling compared the processed samples in order to identify variants that resulted in a VCF file being produced. All parameters were set to their default values.

The obtained genomes had a consistent G+C content ranging from 37.58% to 37.88%, and lengths ranging from 29,766 to 29,776 base pairs (bp). The study provided almost complete genomes for each sample, with a median of 4,179,542 reads and an average median depth of 3,099×, covering 99.36% of the genome (DOI: 10.6084/m9.figshare.26281285).

**Fig 1:**
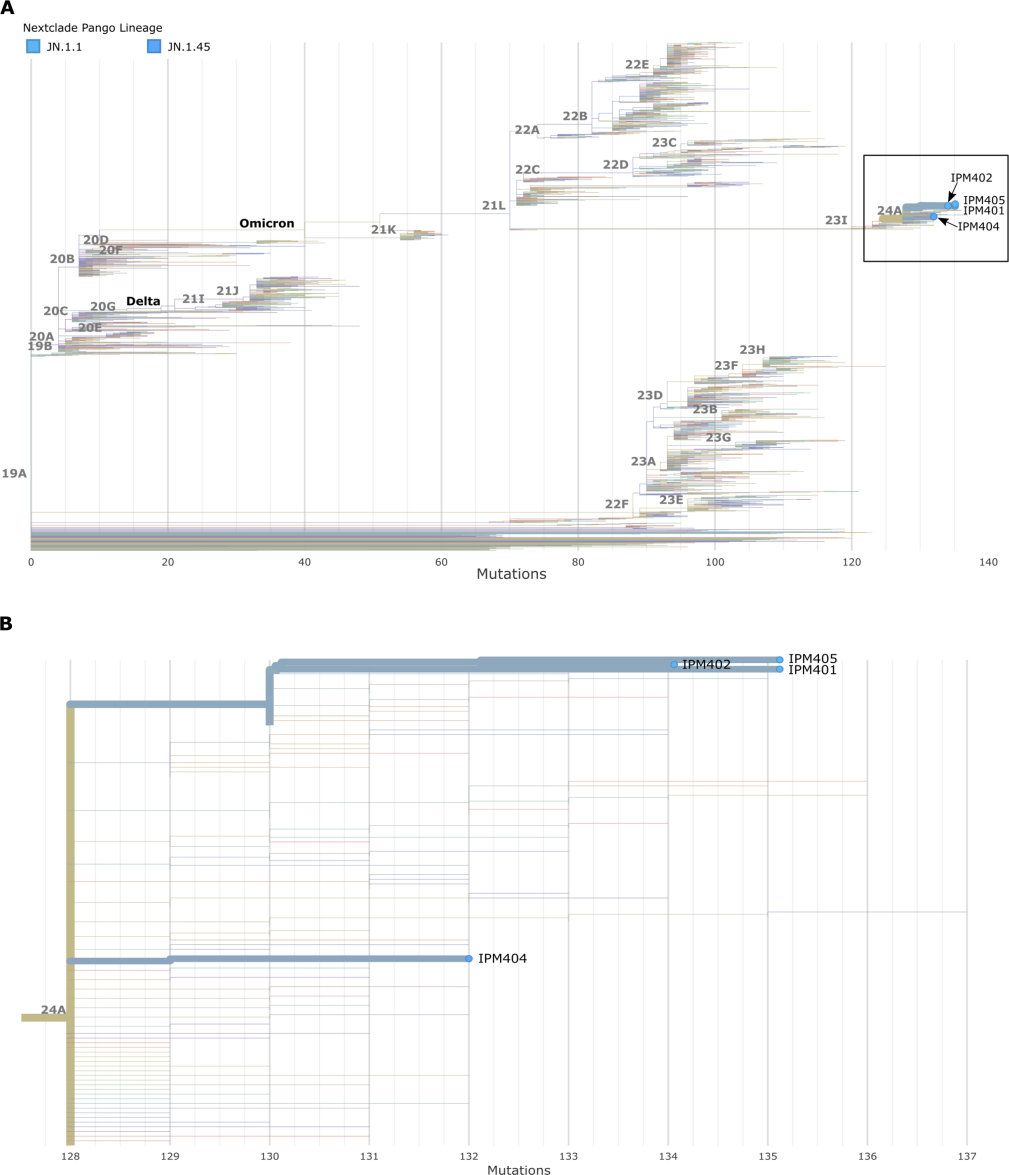
The Nextstrain tree of the SARS-CoV-2 genome sequences. (**A**) Phylogenetic tree of SARS-CoV-2 variants. (B) Phylogenetic position of SARS-CoV-2 JN.1 variant.

Phylogenetic analysis using Nextclade v3.5.0 identified the JN.1 lineage in all four genomes (Fig. 1). Compared to the reference sequence (GenBank MN908947.2), there were 114 variants: 29 in ORF1ab, 64 in spike, 2 in ORF3a, 1 in E, 7 in M, 3 in ORF6, and 8 in N (Table 1).

**TABLE 1:** Types and significance of identified genetic variants

Gene	Nucleotide position	Nucleotide change	Residue change	Effect
ORF1ab	241	-25C>T	No change assigned	upstream_gene_variant
	670	405T>G	Ser135Arg	missense_variant
	897	632C>A	Ala211Asp	missense_variant
	1762	1497C>A	Phe499Leu	missense_variant
	2790	2525C>T	Thr842Ile	missense_variant
	3037	2772C>T	Phe924Phe	synonymous_variant
	3431	3166G>T	Val1056Leu	missense_variant
	3565	3300T>C	Gly1100Gly	synonymous_variant
	4184	3919G>A	Gly1307Ser	missense_variant
	4321	4056C<T	Ala1352Ala	synonymous_variant
	6183	5918A>G	Lys1973Arg	missense_variant
	7842	7577A>G	Asn2526Ser	missense_variant
	8293	8028C>T	Thr2676Thr	synonymous_variant
	8393	8128G>A	Ala2710Thr	missense_variant
	9344	9097C>T	Leu3027Phe	missense_variant
	9424	9159A>G	Val3053Val	
	9534	9269C>T	Thr3090Ile	missense_variant
	10029	9764C>T	Thr3255Ile	missense_variant
	10198	9933C>T	Asp3311Asp	synonymous_variant
	10447	10182G>A	Arg3394Arg	synonymous_variant
	10449	10,184C>A	Pro3305His	missense_variant
	11042	10777G>T	Val3593Phe	missense_variant
	11288	11023-11031del TCTGGTTTT	Ser3675- Phe3677del	disruptive_inframe_deletion
	11727	11462G>A	Arg3821Lys	missense_variant
	11747	11,482C>T	Leu3828Leu	
	12789	12,524C>T	Thr4175Ile	missense_variant
	12815	12,550C>T	Leu4184Leu	synonymous_variant
	12880	12,615C>T	Ile4205Ile	synonymous_variant
	13339	13,074T>C	Asn4358Asn	synonymous_variant
S	21608	46ins TCATGCCGCTGT	insMet-Pro-Leu-Phe	insersion
	21618	56C>T	Thr19Ile	missense_variant
	21622	60C>T	Thr20Thr	
	21624	62G>C	Arg21Thr	missense_variant
	21633	71-79del	Leu24-Pro26delAla27Ser	disruptive_inframe_deletion
	21711	149C>T	Ser50Leu	missense_variant
	21765	203-208del	His60-Val70del	
	21941	56C>T	Thr19Ile	missense_variant
	21987	425G>A	Gly142Asp	missense_variant
	21992	430-432del	Tyrdel	disruptive_inframe_deletion
	22032 22033	470T>C 471C>A	Phe157Ser	missense_variant
	22034	472A>G	Arg158Gly	missense_variant
	22194	632-634del	Asn211delLeu212Ile	disruptive_inframe_deletion
	22200	638T>G	Val213Gly	missense_variant
	22208	646C>T	Leu216Phe	missense_variant
	22295	733C>A	His245Asn	missense_variant
	22353	791C>A	Ala264Asp	missense_variant
	22556	994A>G	Ile332Val	missense_variant
	22577 22578	1015G>C 1016G>A	Gly339His	missense_variant
	22629	1067A>C	Lys356Thr	missense_variant
	22674	1112C>T	Ser371Phe	missense_variant
	22679	1117T>C	Ser373Pro	missense_variant
	22686	1124C>T	Ser375Phe	missense_variant
	22688	1126A>G	Thr376Ala	missense_variant
	22770	1208G>A	Arg403Lys	missense_variant
	22775	1213G>A	Asp405Asn	missense_variant
	22786	1224A>C	Arg408Ser	missense_variant
	22813	1251G>T	Lys417Asn	missense_variant
	22882	1320T>G	Asn440Lys	missense_variant
	22895 22896	1333G>C 1334T>A	Val445His	missense_variant
	22898	1336G>A	Gly446Ser	missense_variant
	22910	1348A>G	Asn450Asp	missense_variant
	22916 22817	1354C>T 1355T>G	Leu452Trp	missense_variant
	22926	1364T>C	Leu455Ser	missense_variant
	22942	1380T>A	Asn460Lys	missense_variant
	22992	1430G>A	Ser477Asn	missense_variant
	22995	1433C>A	Thr478Lys	missense_variant
	23005	1443T>A	Asn481Lys	missense_variant
	23009	1447-1449del	Val483del	disruptive_inframe_deletion
	23012	1450G>T	Glu484Lys	missense_variant
	23018 23019	1456T>C 1457T>C	Phe486Pro	missense_variant
	23055	1493A>G	Gln498Arg	missense_variant
	23063	1501A>T	Asn501Tyr	missense_variant
	23075	1513T>C	Tyr505His	missense_variant
	23222	1660G>A	Glu570Lys	missense_variant
	23271	1709C>T	Ala570Val	missense_variant
	23403	1841A>G	Asp614G	missense_variant
	23416	1854A>T	Thr618Thr	synonymous_variant
	23423	1861C>T	Pro621Ser	missense_variant
	23599	2037T>G	Asn679Lys	missense_variant
	23604	2042C>G	Pro681Arg	missense_variant
	23854	2292C>A	Ans764Lys	missense_variant
	23948	2386G>T	Asp796Tyr	missense_variant
	24378	2816C>T	Ser939Phe	missense_variant
	24424	2862A>T	Gln954His	missense_variant
	24469	2907T>A	Asn969Lys	missense_variant
	24990	3428T>C	Pro1143Leu	missense_variant
	25000	3438T>C	Asp1146Asp	synonymous_variant
	25207	3645T>C	Tyr1215Tyr	synonymous_variant
ORF3a	25584	192C>T	Thr64Thr	synonymous_variant
	26060	669C>T	Thr223Ile	missense_variant
E	26270	26C>T	Thr9Ile	missense_variant
M	26529	7G>C	Asp3His	missense_variant
	26577	55C>G	Gln19Glu	missense_variant
	26610	88A>G	Thr30Ala	missense_variant
	26681	159C>T	Phe53Phe	synonymous_variant
	26709	187G>A	Ala63Thr	missense_variant
	26833	311C>T	Ala104Val	missense_variant
	26858	336C>T	Phe112Phe	
ORF6	27382 27383 27384	181G>C 182A>T 183T>C	Thr61Leu	missense_variant
N	28311 28312	38C>T 39C>T	Pro13Leu	missense_variant
	28362	89-97del	Glu31-Ser33del	disruptive_inframe_deletion
	28881 28882	608G>A 609G>A	Arg203Lys	missense_variant
	28883	610G>C	Gly204Arg	missense_variant
	28958	685C>A	Gln229Lys	missense_variant
	29510	1237A>C	Ser413Arg	missense_variant

## Data Availability

These sequences were deposited at GenBank under the accession numbers PP832907, PP832908, PP832909, and PP832910. The raw reads were deposited at the NCBI Sequence Read Archive (SRA) under the accession numbers SRR29285436, SRR29285437, SRR29285438, and SRR29285439. The data presented in this study are available upon reasonable request from the corresponding author.
